# Comparison of ^99m^Tc-MIBI scintigraphy, ultrasound, and mammography for the diagnosis of BI-RADS 4 category lesions

**DOI:** 10.1186/s12885-020-06938-7

**Published:** 2020-05-24

**Authors:** Hongbiao Liu, Hongwei Zhan, Da Sun

**Affiliations:** Department of Nuclear Medicine, The Second Affiliated Hospital, Zhejiang University School of Medicine, 88 Jiefang Road, Hangzhou, China

**Keywords:** Breast-specific gamma imaging/BSGI, Mammography, Ultrasound, Breast cancer

## Abstract

**Background:**

We sought to determine the diagnostic efficacy of Breast-specific gamma imaging (BSGI) in Chinese women with BI-RADS 4 category lesions and to compare this efficacy to that of ultrasound/mammography.

**Methods:**

We retrospectively analyzed data from 177 women that had undergone BSGI of BI-RADS 4 category lesions originally detected via ultrasound and/or mammography.

**Results:**

Of these 177 cases, 117 (66.1%) were malignant lesions and 60 (33.9%) were benign. The sensitivity, specificity, positive predictive values, and negative predictive values of BSGI were 94.9% (111/117), 78.3% (47/60), 89.5% (111/124), and 88.7% (47/53), respectively. The specificity and positive predictive values for mammography were 48.3% (29/60) and 77.5% (107/138), while for ultrasound they were 53.3% (32/60) and 79.6% (109/137), respectively. The sensitivity and specificity of BSGI for the detection of lesions ≤1 cm in size were 90.9% (10/11) and 88.0% (22/25), respectively, while for breast lesions >1 cm in size these values were 94.3% (100/106) and 71.4% (25/35), respectively. In addition, BSGI sensitivity and specificity values for dense breast tissue were 94.0% (79/84) and 78.0% (39/50), respectively, whereas for non-dense breast tissue these vales were 97.0% (32/33) and 80.0% (8/10), respectively. The sensitivity of BSGI for invasive ductal carcinomas (IDC) and ductal carcinomas in situ (DCIS) was 98.9% (95/96) and 75.0% (9/12), respectively. The tumor to normal tissue ratio of BSGI for malignant lesions was significantly higher than for benign lesions (2.18 ± 1.17 vs 1.66 ± 0.40, *t* = 7.56, *P*<0.05).

**Conclusions:**

These results indicate that BSGI is highly sensitive for the detection of such lesions, achieving good positive/negative predictive values. This suggests that for IDC in particular, BSGI is superior to ultrasound and mammography for the diagnosis of BI-RADS 4 category lesions, although this was less apparent for the diagnosis of DCIS lesions. BSGI exhibited excellent performance in dense breast tissue and for the detection of lesions ≤1 cm in size.

## Background

Rates of cancer have increased by nearly 28% between 2006 and 2016, with breast cancer remaining the most frequent cancer-related cause of death among women [1]. Early-stage breast cancer diagnosis can improve patient survival. Herein we analyzed data pertaining to Breast Imaging Reporting and Data System (BI-RADS) 4 category lesions detected via ultrasound and/or mammography in order to resolve the best approach to accurately diagnosing these lesions. The BI-RADS 4 classification is intended to designate potentially suspicious masses warranting biopsy, with 3 subtypes (4a, 4b, and 4c) of increasing suspicion. These lesions, by definition, do not exhibit the morphological characteristics of breast cancer. However, they have a risk of malignancy that can range from 2 to 95% over time. This variability can lead to the unnecessary biopsy and over-treatment of benign lesions [2]. Breast-specific gamma imaging (BSGI) is a high-resolution radioimaging approach that can be employed to visualize breast tissues using a small field-of-view gamma camera that is confined to the breast region. In this study, we evaluated the diagnostic efficacy of BSGI for the differential diagnosis of BI-RADS 4 lesions identified via ultrasound/mammography and we compared these three approaches in order to evaluate their relative diagnostic utility.

## Methods

### General information

The hospital ethics committee approved this retrospective study. Written informed consent was obtained from each patient. In total, we retrospectively studied 177 patients who were diagnosed and treated at our Hospital (Second Affiliated Hospital of Zhejiang University School of Medicine, Hangzhou, China) between January, 2015 and June, 2018. All analyzed patients had been identified as having BI-RADS 4 category masses based via ultrasound and/or mammography, and had also undergone BSGI at our Hospital. Patients who had previously undergone surgery for breast cancer were not included in this study. After imaging examinations, histopathologic confirmation was performed via surgical excision or core needle biopsy. All patients’ clinical records were reviewed, while clinicopathological characteristics including age, menstrual state, clinical stage, lesion location, tumor size and grade, and histological type were obtained from medical records at our institution.

### Imaging method

Patients were administered 555–740 MBq of ^99m^Tc-MIBI (Shanghai GMS Pharmaceutical Co., Ltd) through the antecubital vein contralateral to the breast lesion. Once 10 min had elapsed following tracer injection, BSGI was conducted. Patients in a sitting position were imaged with a breast-specific gamma camera (Dilon 6800; Dilon Technologies Inc., USA), allowing for the high-resolution bilateral collection of craniocaudal (CC) and mediolateral oblique (MLO) images. The acquisition time for each image was approximately 5 min, and 100,000 counts per image were defined as the minimal range. Two physicians specializing in nuclear medicine interpreted all BSGI images according to the Society of Nuclear Medicine published BSGI operation guidelines for interpreting BSGI imaging results [3]. Positive BSGI tumors were those which were determined to either exhibit a tumor-to-normal tissue ratio (TNR) > 2.09, or to be highly suspicious. Lesions analyzed by ultrasound were determined to be positive based on suspicious appearance suggesting the need for biopsy or removal. Two radiologists interpreted all mammography images based on BI-RADS classification.

### Statistical analysis

The sensitivity, specificity, positive predictive values, and negative predictive values for BSGI, ultrasound, and mammography were determined. The relative efficiencies of BSGI, ultrasound, and mammography, as well as the semi-quantitative indicators thereof, were calculated and compared via χ^2^ tests. SPSS v20 was used for all statistical testing, with *P* < 0.05 as the significance threshold.

## Results

### Ultrasound and mammography

Our study included 177 patients, of whom 135 (76.3%) were diagnosed with BI-RADS 4 category lesions by ultrasound, 127 (71.8%) cases were diagnosed as having BI-RADS category 4 lesions via mammography, and 87 (49.1%) were diagnosed via both of these approaches.

### Pathologic results

Of the 177 female patients, 117 (66.1%) were ultimately found to have malignant tumors. Of those with malignant lesions, the average patient age was 53.5 years old (range: 23–89). Detected malignant lesions included invasive ductal carcinomas (IDC, *n* = 96), ductal carcinomas in situ (DCIS, *n* = 12), invasive lobular carcinomas (*n* = 2), malignant phyllodes tumors (n = 2), and others (*n* = 5). The remaining 60 (33.9%) cases proved to be benign. Of patients with benign lesions, the average age was 47.2 years old (range: 19–74). Benign lesions included fibroadenomas (*n* = 31), adenosis (*n* = 17), intraductal papillomas (*n* = 7), and others (*n* = 5).

### Diagnosis via BSGI, ultrasound, and mammography

**Fig. 1:**
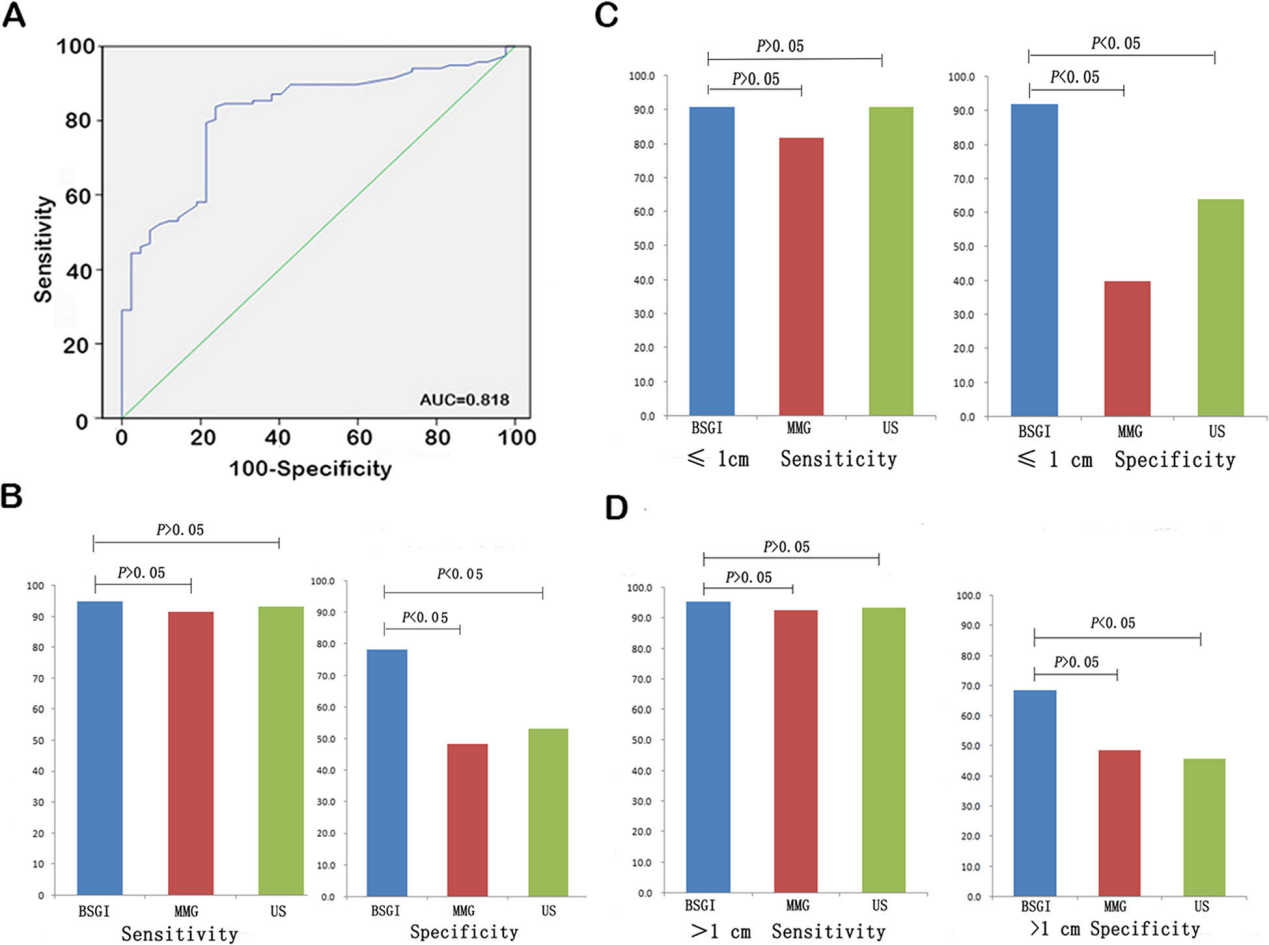
The diagnostic use of BSGI, MMG, and US for the detection of breast lesions. **a**. Am ROC analysis was used to determine the TNR cut-off value for the detection of breast cancer. **b**. Sensitivity of BSGI, MMG, and US for detecting breast cancer when used to analyze BI-RADS 4 category lesions. **c**. Sensitivity of BSGI, MMG, and US for detecting breast cancers ≤1 cm in size. **d**. Sensitivity of BSGI, MMG, and US for detecting breast cancers >1 cm in size

The TNR cut-off for sensitivity and specificity was 2.09 (Sensitivity: 62.4%, Specificity: 78.6%) (Fig. 1a). We found that there was a statistically valid correlation between lesion malignancy and TNR ratio. The TNR ratio for the malignant group was 2.18 ± 1.17, while the TNR ratio for the benign group was 1.66 ± 0.40 (*t* = 7.56, *P*<0.05). The sensitivity of BSGI, mammography, and ultrasound for malignant lesion detection reached 94.9, 91.5, and 93.2%, respectively. BSGI had the greatest specificity at 78.3%, compared to mammography (48.3%, *P* < 0.05) and ultrasound (53.3%, *P* < 0.05) (Fig. 1b). Sensitivity and specificity findings for these imaging approaches are shown in Table 1

**Table 1:** Comparison of BSGI, mammography, and ultrasound sensitivity and specificity for BI-RADS 4 lesions

Approach	Sensitivity	Specificity	PPV	NPV
BSGI	94.9 (111/117)	78.3 (47/60)	89.5 (111/124)	88.7 (47/53)
Mammography	91.5 (107/117)	48.3 (29/60)*^a^	77.5 (107/138)*^b^	74.4 (29/39)
Ultrasound	93.2 (109/117)	53.3 (32/60) *^a^	79.6 (109/137) *^b^	80.0 (32/40)

*PPV* positive predictive value, *NPV* negative predictive value

^*a^BSGI to Mammography, *P* < 0.05, to Ultrasound, *P* < 0.05

^*b^BSGI to Mammography, *P* < 0.05, to Ultrasound, *P* < 0.05

.

Malignant lesions were between 3 mm and 74 mm, with 11 lesions being smaller than 10 mm. Benign lesions were between 2 mm and 44 mm, with 25 lesions being smaller than 10 mm. The determination of tumor lesion size via surgical excision was based on pathological results. Tumor lesion size via focus of puncture was based on the results of ultrasound/ mammography. The sensitivity of BSGI was similar to that of ultrasound for lesions≤1 cm, whereas BSGI specificity was highest at 88.0%, as compared to mammography (40.0%, *P* < 0.05) and ultrasound (64.0%, *P* < 0.05) (Fig. 1c). For lesions > 1 cm in size, the specificity of BSGI was 71.4% as compared to ultrasound at 45.7% (*P* < 0.05) (Fig. 1d).

**Fig. 2:**
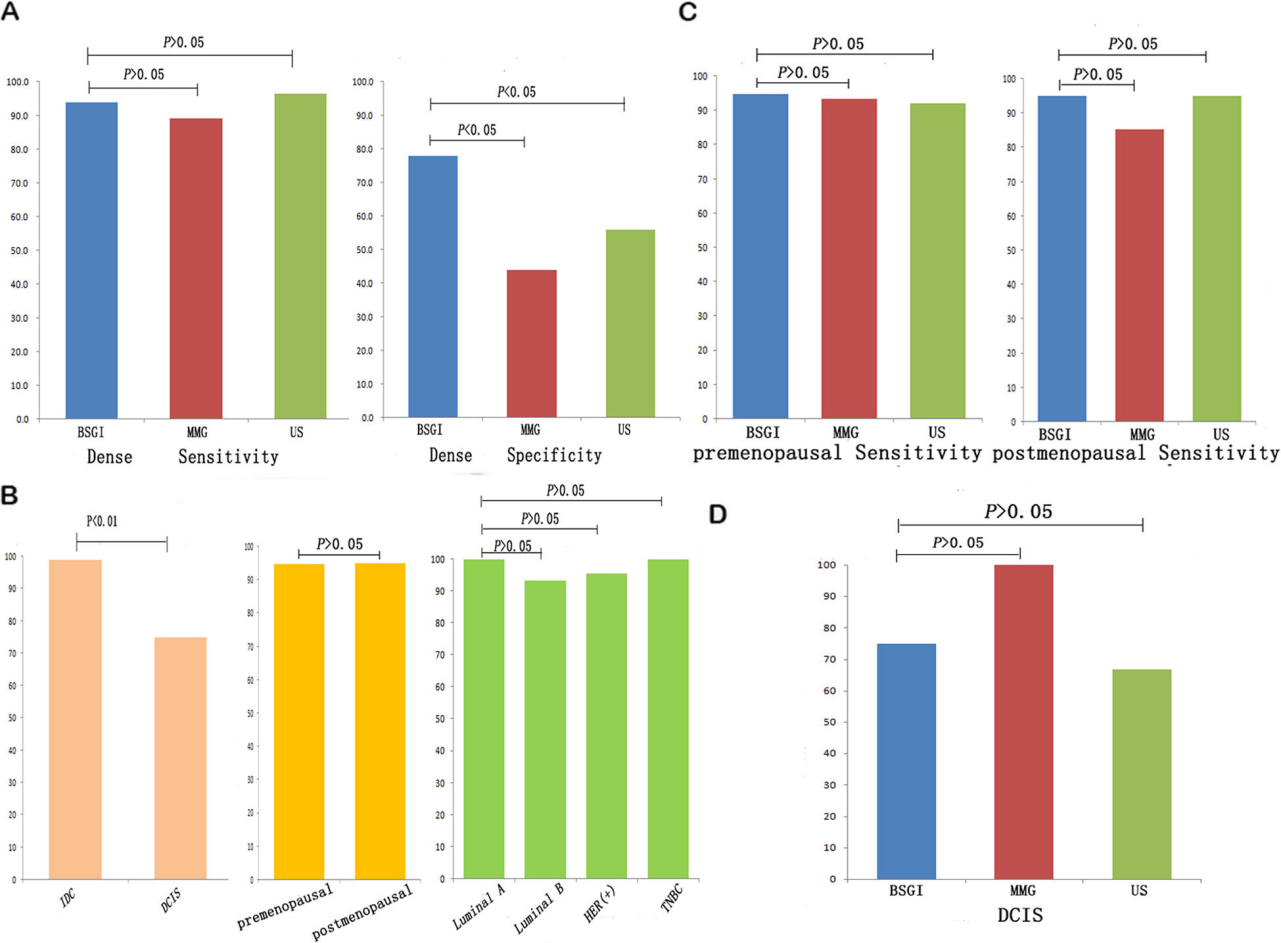
The sensitivity of BSGI, MMG, and US in different breast cancercontexts. **a**. The sensitivity and specificity of BSGI, MMG, and US in the detection of breast cancer within dense tissue. **b**. The sensitivity of BSGI, MMG, and US in the detection of breast cancer in premenopausal and postmenopausal women. **c**. The sensitivity of BSGI, MMG, and US for the detection of different breast cancer molecular subtypes. **d**. The sensitivity of BSGI, MMG, and US for the detection of DCIS

The American College of Radiology BI-RADS classifications are intended to offer a standardized means of reporting on the density of mammographic findings. Of these 177 subjects, 134 (75.7%) were shown to have dense breast tissue with a BI-RADS density category of 3 or 4 upon mammographic assessment. In these women, BSGI specificity was highest at 94.0%, as compared to mammography (44.0%, *P* < 0.05) and ultrasound (56.0%, *P* < 0.05) (Fig. 2a). In pre- or post-menopausal women, BSGI sensitivity was not greater than that of mammography or ultrasound, nor was there a significant difference in sensitivity between the pre- and post-menopausal groups (Fig. 2b). With respect to cancer types, the sensitivity for the detection of IDC by BSGI was superior to that of DCIS (*P* < 0.05). The sensitivity of BSGI was equivalent between luminal-A, luminal-B, HER2 (+), and triple-negative breast cancers (Fig. 2c). BSGI sensitivity for the detection of DCIS was similar to that of mammography or ultrasound (Fig. 2d)

.

### False-positive and -negative BSGI findings

This study revealed 13 false-positive and 6 false-negative BSGI findings, the pathology findings of which are shown in Table 2

**Table 2:** BSGI false-positive and false-negative rates in the diagnosis of BI-RADS 4 category lesions

False-positive		False-negative	
Classification	Number	Classification	Number
Sclerosing Adenosis	6	**Ductal carcinomas in situ**	3
Fibroadenoma	3	**Invasive ductal carcinomas**	1
Usual ductal hyperplasia	2	**Tubular carcinoma**	1
Intraductal papilloma	1	**Mucinous carcinoma**	1
Chronic inflammation	1		

.

## Discussion

**Fig. 3:**
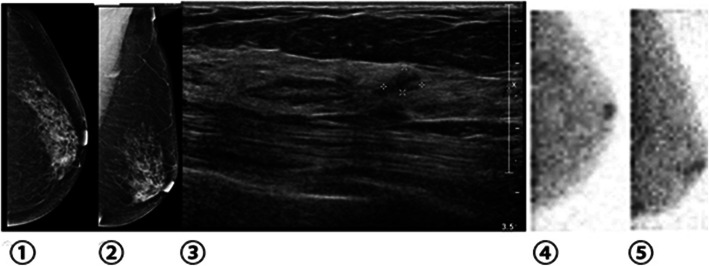
Breast tissue images from a woman. (①②)Mammography (LCC, LMLO) demonstrated a cluster of amorphous micro-calcifications in the upper outer quadrant area, BI-RADS 4B; ③Ultrasound demonstrated a hypoechoic lesion of the left breast, 0.6 cm*0.4 cm, BI-RADS 4A; ④⑤BSGI was negative and the tissue was shown to be normal; Pathology demonstrated this lesion to be a typical ductal hyperplasia

Several studies have shown that BSGI is highly sensitive for the detection of breast cancer in women with dense and non-dense breasts, and that it is an effective adjunct imaging modality in women with both dense and non-dense breasts [4]. The value of this approach is highest for the detection of small carcinomas and multifocal/ multicentric disease [5]. In this study, we explored the diagnostic efficacy of BSGI for differentiating between malignant and benign BI-RADS 4 lesions relative to that of ultrasound or mammography in Chinese women. Our results demonstrate that BSGI is well-suited to the diagnosis of suspicious lesions. Mammography is one of the primary approaches to breast cancer screening, achieving a high degree of sensitivity for the detection of breast cancer in most patients, and with its clinical implementation having led to a decline in breast cancer death rates [6]. While mammography has become the standard means of breast cancer screening, its accuracy and sensitivity are influenced by breast density and the presence of scar tissue, with the degree of density leading to sensitivity values ranging from 85 to 68%. Breast density is also strongly correlated with the risk of breast cancer [7–9]. Among Chinese populations, approximately 75% of women have heterogeneously or extremely dense breasts, limiting the utility of mammography- based screening. This is particularly true in young populations in which there is a trend towards increased breast cancer incidence, and as such an alternative method of breast cancer detection is needed for this population. Unlike mammography, the sensitivity of the BSGI is not affected by breast tissue density, prosthesis implantation, structural deformation, scarring, or radiation therapy. Yu et al. [10] reported that the diagnostic specificity of BSGI for breast cancer was high (83.2%), and that in women with dense breast tissue BSGI was superior to mammography. Kessler et al. [11] reported on 93 cases of BI-RADS 4 breast lesions detected via mammography, with a positive rate of biopsy of 14%. Some special pathological types of breast cancer, such as early-stage invasive lobular carcinomas, are difficult to detect via conventional imaging approaches, whereas the detection rates for such carcinomas by BSGI are over 90% [12]. Spanu et al [13] evaluated the utility of breast-specific gamma camera (BSGC) scintigraphy for DCIS identification, achieving a 93.9% sensitivity, which was slightly higher than that achieved via mammography while allowing for a better assessment of the extent of local disease. Consistent with this, in the present study, 2 invasive lobular carcinoma lesions were correctly diagnosed by BSGI (Fig. 3).

**Fig. 4:**
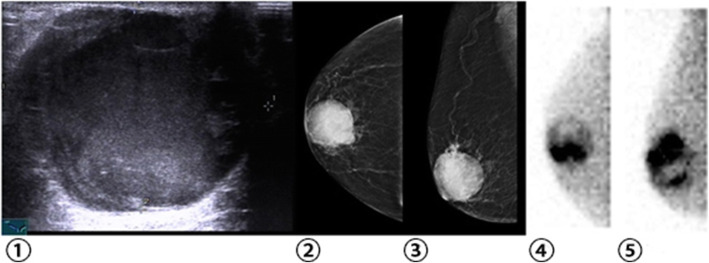
Breast images from an old woman. (①)Ultrasound demonstrated a cystic solid mass in the upper outer region of the right breast, 4.4 cm*3.5 cm; (②③)Mammography demonstrated a high density shadow located in the upper outer region of the right breast, 44 mm*46 mm, BI-RADS 4B; (④⑤)BSGI revealed a focal region of increased radiotracer uptake in the right breast, TNR = 3.59; Pathology identified a 74-mm invasive ductal carcinoma

Ultrasound, as a first-line examination used to differentiate between benign and malignant breast lesions, offers the advantages of being non-invasive, convenient, and efficient. It has been widely used for the BI-RADS classification of breast lesions in the clinic. However, the utility of ultrasound for breast lesions has been varied for some types of lesions, with a lack of specificity for some, or higher false-positive detection rates [14]. Patients with mammary intraductal papillomas typically present with nipple discharge and lumps in the area, which are characterized by a hypoechoic lesions upon ultrasound examination. Tadwalkar et al. [15] retrospectively analyzed BSGI findings for 139 females with invasive carcinoma. The efficiency of the BSGI-mediated diagnosis of breast cancer is related to tumor differentiation and size. In this study, for lesions≤1 cm in size the sensitivity of BSGI was similar to that of ultrasound, while the specificity of BSGI was superior to ultrasound. In addition, 4 intraductal papillomas were analyzed by ultrasound were determined to be positive based on highly suspicious appearance, yet were correctly determined to be negative via BSGI (Fig. 4).

**Fig. 5:**
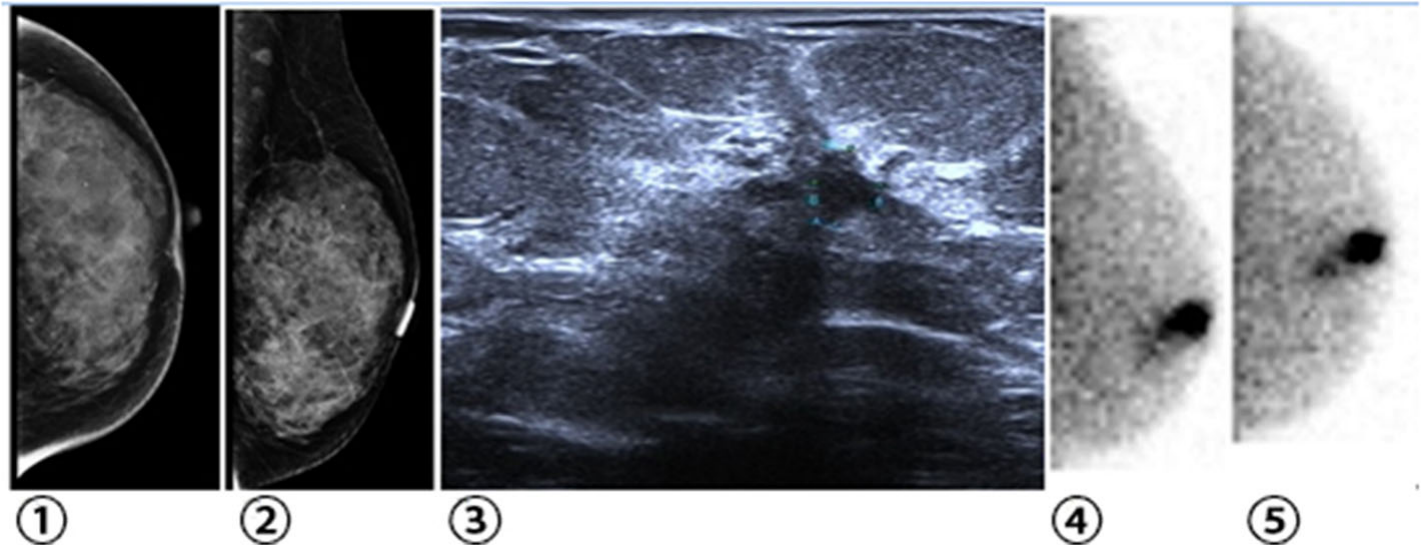
Breast images from a woman. (①②) No mammographic evidence of dense breast abnormalities; (③)An ill-defined hypoechoic nodule was detected via ultrasound in the left breast, 0.6 cm*0.5 cm, BI-RADS 4B; (④⑤) BSGI revealed a focal region of increased radiotracer uptake in the left breast, TNR = 3.21; Pathology confirmed the presence of a 7 mm invasive ductal carcinoma

Ultrasound and mammography are anatomical approaches to detecting breast cancer. Nuclear medicine approaches that instead rely upon the physiological characteristics of tumors are increasingly common. The detection of breast cancer can be improved by using physiological imaging as an auxiliary imaging modality. BSGI is a functional imaging test that offers better sensitivity than does traditional planar scintigraphy, particularly in patients with dense breast tissue, and it is also better for the detection of subcentimeter (< 1 cm) lesions [10]. The radioactive tracer ^99m^Tc-MIBI, after injection into the human body, enters into mitochondria. The number of mitochondria in cells is closely related to cell activity, and as malignant cells have a higher metabolic rate they have more mitochondria. As a result, the uptake of ^99m^Tc-MIBI in cancer cells is greater than that in surrounding normal tissue. In vitro experiments show that tumor cells absorb ^99m^Tc-MIBI at a rate > 50% higher than normal cells [16]. Kim et al. [17] conducted a retrospective analysis of 520 suspected breast cancer patients and found that, when using a ^99m^Tc-MIBI imaging approach, the TNR in the malignant group was 2.00 ± 1.88, which was significantly higher than in the benign group (0.60 ± 0.70). In our study, the TNR in the malignant group was 2.18 ± 1.17, which was also significantly higher than that in the benign group (1.66 ± 0.40). However, BSGI appears to be an ineffective diagnostic tool for the detection of breast cancer axillary lymph node metastasis. In a study by Spanu et al. [18], the authors analyzed 76 consecutive breast cancer patients, of whom 28 had axillary lymph node metastases, and they achieved true positive results via BSGU in only 7 patients (sensitivity: 25%), whereas SPECT achieved true positivity in 23 of 28 cases (sensitivity: 82.1%) and Pinpole-SPECT achieved true positivity in 25 of 28 cases (sensitivity: 89.3%) (Fig. 5).

Other modes of examination such as shear-wave elastography (SWE) and breast MRI have also been used in an effort to improve the accuracy of breast cancer diagnosis. SWE is a quantitative imaging technique widely used during breast imaging. Because breast malignant tissues are harder than normal tissues, SWE not only can locally quantify tissue hardness but also provide histological information. Yoon et al. [19] conducted an analysis of 199 consecutive women, demonstrating that SWE had significantly false-positive rates than false-negative rates. Size, breast thickness, and depth all influenced SWE findings. Bilimoria et al. [20] reported that breast MRI is the most sensitive imaging modality for detecting breast cancer, and found that it may reveal breast cancers which are difficult to detect via physical examination, mammography, or ultrasound. However, breast MRI not only has a considerable false-positive rate but also has the potential to enlarge the apparent size of the tumor in question. As a result, women assessed in this manner may undergo unnecessary mastectomy. Finally, the conventional use of breast MRI is limited by the cost of this analytical approach. In addition, the continued development of digital x-ray systems has enabled further techniques such as contrast mammography to overcome the limitations of mammography, and similar novel approaches are now being investigated. Fallenberg [21] et al. reported that contrast mammography is as accurate as MRI. However, this is an invasive technique, and the injection of contrast agents is not acceptable or suitable for breast cancer screening efforts.

^99m^Tc-MIBI, as a non-specific tumor imaging agent, can yield false-positive results for certain benign hyperplastic lesions, decreasing its diagnostic performance. Fibrocystic breast disease, fibroadenomas, and breast benign hyperplasias were the most common types of false-positive lesions detected by BSGI [22]. Malignant breast tumors can promote local angiogenesis, and some benign growths may mimic this activity, although in a manner not equivalent to that of malignant lesions. For example, some intraductal papilloma, inflammatory lesions, fibroadenomas, or adenopathies have abundant blood supplies. Inflammatory lesions also have the characteristics of inflammatory cells, with peripheral irregular infiltration of surrounding tissues. Weight et al. [23] reported that BSGI resulted in a higher rate of change in the management of patients (109/119) as compared with ultrasound (71/119), and found BSGI performed better than ultrasound in terms of both positive predictive value and accuracy. Overall, this study identified a total of 13 lesions with false-positive findings when analyzed via BSGI.

## Limitations of BSGI

Despite its promise, there are certain limitations to the use of BSGI for breast imaging. First, during BSGI, patients were injected with ^99m^Tc-MIBI, which is a radiopharmaceutical (555-740 MBq), leading patients to be exposed to 6.29–9.44 mSv of radiation on average [17, 24]. However, a lower injected dose of 99mTc-MIBI may be acceptable for such imaging. In a study by Rhodes et al. [25], when used to supplement screening mammography, BSGI conducted using 300 MBq of ^99m^Tc-MIBI for screening (effective dose: 2.4 mSv) yielded a supplemental cancer detection rate of 8.8 per 1000 women with mammographically dense breasts, which was equivalent to the results obtained using a higher 740 MBq dose of ^99m^Tc-MIBI, increasing the rate of cancer detection in women with mammographically dense breasts by 7.5 per 1000 screened. Patients who have suspicious lesions or dense breast tissue may be recommended for BSGI, necessitating a longer acquisition time and lower radiotracer doses. Second, as this is a planar test there is a risk of improper positioning [15, 26]. Finally, BSGI was also a relatively insensitive approach for axillary lymph node detection.

## Conclusion

In summary, BSGI offers good diagnostic efficacy for the differential diagnosis of BI-RADS 4 category breast lesions identified via ultrasound and/or mammography. BSGI was a highly efficient means of detecting breast lesions in dense breast tissues and lesions ≤1 cm in size. These results demonstrated that BSGI may be useful as an auxiliary imaging modality for detecting suspicious lesions, and can reduce the need for unnecessary biopsy and surgical procedures, further decreasing rates of misdiagnosis, and improving the accurate diagnosis of breast cancer.

## Data Availability

The data involved in the current study are available upon request. Anyone who is interested in the information should contact hbliu@zju.edu.cn.

## Abbreviations

BSGI: Breast specific gamma imaging
BI-RADS: Breast imaging reporting and data system
IDC: Invasive ductal carcinomas
DCIS: Ductal carcinomas in situ
TNR: Tumor-to-normal tissue ratio
PPV: Positive predictive value
NPV: Negative predictive value
SWE: Shear-wave elastography
